# Converging Development of English as Foreign Language Listening and Reading Comprehension Skills in German Upper Secondary Schools

**DOI:** 10.3389/fpsyg.2020.01116

**Published:** 2020-06-01

**Authors:** Christian Spoden, Jens Fleischer, Michael Leucht

**Affiliations:** ^1^German Institute for Adult Education – Leibniz Centre for Lifelong Learning, Bonn, Germany; ^2^Department of Instructional Psychology, University of Duisburg-Essen, Essen, Germany; ^3^IPN – Leibniz Institute for Science and Mathematics Education, Kiel, Germany

**Keywords:** listening comprehension, reading comprehension, receptive language skills, upper secondary education, cross-lagged panel design

## Abstract

Receptive skills in English as a second language are important for students on the verge of entering higher education as this student group (aged 17–19) is expected to apply English regularly in their later life. Previous research in this age group in Germany already implied an increasing overlap between both skills in this age group, although robustness of this effect across student groups with different learning experiences was not tested. We used language assessment data collected from upper secondary schools (i.e., from 17 to 19-year-old students) in Germany to compare correlations at the beginning and the end of upper secondary education in groups of students from (1) language-related versus non-language-related study profiles and (2) from students with frequent versus less frequent self-reported English-language out-of-school learning activities. In all of these groups, correlations were increasing, indicating converging skills in upper secondary education. The results are discussed in terms of implications for current theories of language research.

## Introduction

In a globalized world, there are few doubts about the importance of English-language skills for students who accomplish upper secondary and possibly higher education. English-language skills are crucial in higher education as a large amount of documented research is only available in English. There is also an increasing number of degree programs, lectures, and training courses held in English outside of English-speaking countries. For example, the number of “English-only” degree programs rose from 391 in 2005 to 1017 in 2015 in Germany (Balzer, 2015). English conversational skills are equally important for students who enter a profession directly after upper secondary school, as jobs addressing the more highly trained most often require advanced to excellent (business) skills in English as lingua franca and common corporate language for internal communication (e.g., Nickerson, 2005; Swift and Wallace, 2011). Advanced skills in English as a second language (ESL) and especially receptive skills, namely listening and reading comprehension, are thus counted among the key competencies necessary for educational and vocational achievement. Age groups on the verge of entering higher education have still been widely overlooked in language assessment research. The effects of different learning opportunities might become noticeable in this age group as schools offer different “study profiles” in upper secondary education (e.g., Leucht et al., 2015), which imply a different course selection in preparation for the final secondary-school examinations (A levels). Students in this age group also engage differently in English-language-related activities outside school. Thus, this study investigated the impact of various in- and out-of-school learning opportunities that arise from different study profiles and different English-language-related activities (i.e., primarily media consumption) on the associations between English-language listening and reading comprehension skills at the beginning and the end of upper secondary schooling.

## Theoretical Background

### Listening and Reading Comprehension as Substantially Associated Skills in L1 and L2 Language Acquisition

Most theories of comprehension recognize differences in the modality of the input, although the importance of such differences in the comprehension process is not accentuated and little information is given on how the comprehension is differently affected by input modalities. McNamara and Magliano (2009) reviewed seven comprehension theories and stated that none of these theories had described differences between modalities in a detailed manner or explained the integration of modalities. Aryadoust (2017) recently proposed an integrated theory of comprehension where modality-specific processes were incorporated as “pre-comprehension.” The theory acknowledges that in these pre-comprehension processes of word recognition (processes of segmentation and selection, recognition and lexical access) listeners are disadvantaged as the auditory input fades away in few seconds while readers can always regress to previous parts of the text. The theory also assumes that later process of comprehension, which involve generation of mental meaning and evolving mental imagery (selection) as well as the following stage of integration, where inferencing and elaboration are activated and meaning is assigned to the textual representations, are taken to be common between listening and reading comprehension. Although processes of perception and recognition were not explicitly integrated in earlier theories of comprehension, all comprehension theories still notice that there is a substantial level of overlap (i.e., a correlation) between listening and reading comprehension; this was verified in several psychometric analyses (e.g., Jeon and Yamashita, 2014; Wolf et al., 2019).

The development of listening and reading comprehension in a second language (L2) differs from the development in the mother tongue (L1), where auditory comprehension skills are naturally acquired in early childhood and the acquisition of reading skills usually originates in elementary school. Still, theories like the simple view of reading explain (early) reading comprehension in L1 and L2 as a product of decoding and listening comprehension; a larger number of studies provided evidence for the theory in L2 learning (e.g., Yagoub-Zadeh et al., 2012; Gottardo et al., 2018). On the other hand, L2 listening comprehension, especially in early stages of L2 learning, involves different tasks for the language learner and requires high levels of attention and working memory, as the speed and pronunciation of an authentically spoken foreign language is hardly controllable by the listener (e.g., Vandergrift, 2007; Vandergrift and Baker, 2015). In contrast, the training of early L2 reading comprehension is facilitated by the persisting availability of the text. At later stages of language acquisition, listening comprehension potentially benefits from overlapping subskills (e.g., an enlarged range of vocabulary and grammar knowledge). This might explain higher gains in listening comprehension compared to reading comprehension at the end of secondary schooling (Leucht et al., 2015). However, out-of-school learning opportunities may also contribute to these differences. It has also been shown for some time that out-of-school training of reading fosters reading comprehension (e.g., Watkins and Edwards, 1992; Pfost et al., 2013). Listening comprehension in later adolescence notably benefits from implicit training through out-of-school English-language media consumption in closed-captioned television (Huang and Eskey, 1999), gaming (Sylvén and Sundqvist, 2012), or new educational technology for leisure activities (Liu et al., 2017).

### The Development of Listening and Reading Comprehension in German Upper Secondary Schools

Leucht et al. (2015) analyzed the development of both L2 (English) competencies in upper secondary schools in Germany by means of language assessment data. They found that the development of reading comprehension was slowing down (*d* = 0.15 to *d* = 0.38, depending on different study profiles) but high learning gains were identified in listening comprehension (*d* = 1.04 to *d* = 1.35). The results were moderated by school profiles, with stronger learning gains found in schools with a language-related study profile, which involves additional instruction courses in (foreign) languages. In the studied German federal state of Schleswig-Holstein, schools can offer up to five study profiles (languages, aesthetics with music and arts, sport, science, and social sciences). In the language-related profile, L1 and L2 are usually complemented by a third language (second foreign language) taught with 4 h of instruction a week and two additional foreign languages taught with 3 h a week. In contrast, in the science study profile, as an example for a non-language-related profile, three sciences are taught with increased expenditure of time, but no other languages are taught in non-language-related study profiles besides German classes and English classes. Correlations between listening and reading comprehension in the study by Leucht et al. (2015) increased over a two-year period up to the end of upper secondary education. Investigating this finding in more detail was beyond the scope of their analyses and, thus, Leucht et al. did not study how these skills developed in different student groups and whether the convergence of both skills was a consistent pattern in all groups. Yet, the finding is in need of some further investigation.

## Research Questions

Considering previous results of Leucht et al. (2015) on the converging development of listening and reading comprehension in this age group, the robustness of this effect across students with different in- and out-of-school learning opportunities was investigated in this study. Varying associations between learning-process related variables (e.g., cognitive ability) or other individual characteristics (e.g., gender, cultural capital) and receptive skills depending on different structural assumptions on language skills have already been demonstrated in younger age groups (Hartig and Höhler, 2008). The structure and the associations between both skills were also analyzed within and between classrooms (Höhler et al., 2010) and across different age groups (Tilstra et al., 2009), as examples for different learning settings. Thus far, this research did not involve the relevant group of students from upper secondary schools, although the potential learning trajectories actually grows especially in this age group with study profiles, English language media exposure or other types of possible in- and out-of-school learning opportunities. It is rather plausible to assume that different learning opportunities not only affect achievement in ESL learning (see above) but also the associations between language skills. Thus, the robustness across different learning-related variables becomes important in studying the development of the correlation of listening and reading skills in these different groups.

Information on study profiles was used to analyze effects of different curricular activities and subjective student ratings on activities related to the English language (e.g., English-language media consumption, engagement in English-language conversation on holidays, etc.) in self-reports were used as a proxy variable for the various out-of-school learning opportunities of the students. Based on this information, the study addressed the following two research questions:

1. Do L2 listening and reading comprehension skills in upper secondary education converge in groups of students from non-language-related and language-related study profiles (RQ1)?
2. Do L2 listening and reading comprehension skills in upper secondary education converge in groups of students with and without language-related out-of-school learning experiences (RQ2)?

Quantitative analyses were carried out, making use of the data from Leucht et al. (2015) from a language assessment administered in the German federal state of Schleswig-Holstein, to study these research questions.

## Method

### Sample

To investigate the first research questions, longitudinal data from Grades 11 (T1) and 13 (T2) provided by *N* = 1171 students (*n* = 228 with a language-related study profile) nested in 68 classes from 17 schools in the German federal state of Schleswig-Holstein were analyzed. To investigate the second research question, a subsample (*n* = 550; including 20 students with missings on the scale) from the original sample that had responded to a supplementary survey involving questions on out-of-school learning opportunities (see below) was analyzed.

The interval between the measurement points was 27 months; the measurement points marked the beginning and the end of upper secondary education in Germany (“Oberstufe”). Population weights were computed for each student, so that the real number of students in this grade in the state of Schleswig-Holstein could be approximated in both analyses.

### Instruments

Listening comprehension and reading comprehension skills were measured by means of standardized test instruments. The assessment framework for English listening comprehension and reading comprehension was based on the German Educational Standards (see Rupp et al., 2008), which were themselves based on the Common European Framework of Reference for Languages (Council of Europe, 2001). The items were designed by trained item developers, piloted and optimized by item elimination before the assessment took place. The listening comprehension test comprised 118 items at T1 (*rel*_*PV*_ = 0.75) and 32 items at T2 (*rel*_*PV*_ = 0.82). The reading comprehension test comprised 133 items at T1 (*rel*_*PV*_ = 0.77) and 42 items at T2 (*rel*_*PV*_ = 0.82), administered in complex test designs. All items administered at T2 had previously been used at T1 in order to facilitate the linking of both measurement points on the same scale.

An additional questionnaire was administered to assess out-of-school learning activities as part of a supplementary survey. The questionnaire comprised eight items concerned with either listening to or reading English content in leisure time. Sample items are “I listen to audio books in English.” and “I read books, newspapers, or magazines in English.” (translation by authors). The students responded on a five-point scale with response options ranging from *never* to *more than five times*. The items on English-language-related activities obtained an internal consistency of α = 0.84, indicating that these activities are part of consistent behavioral patterns.

### Data Analysis

In the first step of analysis, the language assessment data were scaled according to a Rasch model (e.g., Spoden and Fleischer, 2019), and plausible values were estimated for both measurement occasions. Plausible values (PVs; e.g., Wu, 2005) estimation is a statistical technique to approximate population characteristics in assessments by random draws from an empirically derived ability distribution. The Rasch model involved a latent regression with several factorized covariates incorporated (e.g., cognitive ability, HISEI, and gender). Item parameters were constrained to the same values for common items at T1 and T2 in order to establish a common metric. The PVs were also rescaled to the metric of the German Educational Standards (*M* = 500, *SD* = 100). The ConQuest software package (Adams et al., 2015), Version 4.0, was used to estimate the latent regression Rasch models.

To investigate the first research question, correlations of both skills at T1 and T2 (PVs) were computed in the two groups of students from language- and non-language-related study profiles. To investigate the second research question, the continuous variable of out-of-school learning experiences was dichotomized by means of a median split. Correlations were computed for students with below and above median scores on this variable. It was also checked in each of these groups separately whether constraining the correlations of listening and reading comprehension at T1 and at T2 to be equal deteriorated model fit. Afterward, it was checked whether constraining the T1 and T2 correlations in both groups of analysis to equal parameters yielded a more parsimonious model fit. The PV correlations were estimated in the Mplus 8.0 software package (Muthén and Muthén, 1998–2017) with sampling weights incorporated into the model estimation and results pooled according to the rules by Rubin (1987).

## Results

### Descriptive Measures on Learning Gains

**FIGURE 1:**
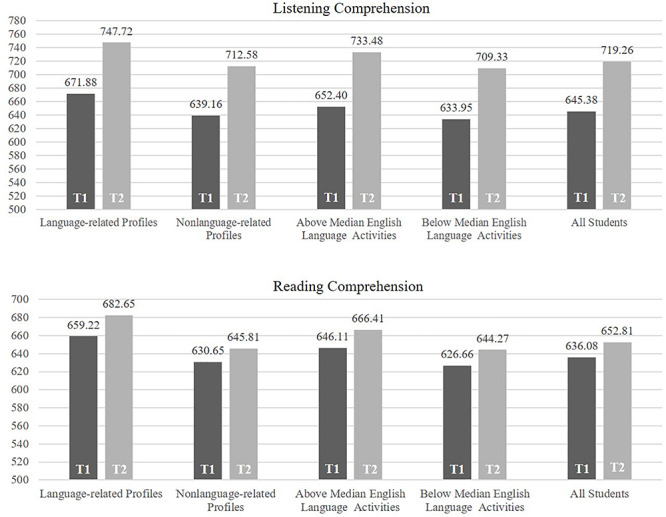
Listening comprehension and reading comprehension for students in the language-related and the non-language-related study profiles, for students with above and below median English language-related extracurricular activities, and for all students at two measurement occasions (T1 = Measurement Occasion 1, T2 = Measurement Occasion 2).

The descriptive measures on gains in English-language receptive skills over the two-year period in the five groups are given in Figure 1. Gains were stronger for listening comprehension compared to reading comprehension, in language-related study profiles compared to non-language related study profiles and with students with higher levels of self-reported out-of-school learning experiences compared to students with lower levels.

### Results for Research Question 1

The results on the convergence of both competencies in language- and non-language-related study profiles over the course of about two years are given in Table 1 (upper part). These results revealed in both groups that constraining the correlations between listening and reading comprehension at T1 and T2 to equal parameter values did not provide a more parsimonious model. Comparing an unconstrained multiple-group model (AIC = 51,623.16, BIC = 51,770.06, BIC_*adj*_ = 51,677.94) with a constrained model, where correlations between listening and reading comprehension were constrained to be equal across both groups of students from language- and non-language-related study profiles (AIC = 51,625.16, BIC = 51,761.93, BIC_*adj*_ = 51,676.17; χ^2^(2) = 6.00, *p* < 0.05), gave mixed results. Following AIC and the likelihood ratio, equally growing correlations of different size in the two groups of students from language-related study profiles from 0.52 at T1 to 0.69 at T2 and the group of non-language-related study profiles from 0.56 at T1 to 0.77 at T2 were to be assumed.

**TABLE 1:** Model fit in group-specific analyses with unconstrained correlations and correlations at T1 and T2 constrained to be equal.

	**Unconstrained correlations**			**Constrained correlations**			**χ^2^(1)**
	**AIC**	**BIC**	**BIC_*adj*_**	**AIC**	**BIC**	**BIC_*adj*_**	
Language-related study profiles	9761.73	9809.74	9765.37	9769.15	9813.73	9772.53	9.43**
Non-language-related study profiles	40, 719.00	40, 786.89	40, 742.43	40, 797.18	40, 860.22	40, 818.93	80.18***
Below median out-of-school Learning experiences	10, 735.50	10, 784.69	10, 740.31	10, 752.33	10, 798.01	10, 756.80	18.83***
Above median out-of-school Learning experiences	12, 120.89	12, 171.88	12, 127.49	12, 132.12	12, 179.46	12, 138.24	13.22***

**p < 0.01. ***p < 0.001.

### Results for Research Question 2

The results on correlations of both competencies in language- and non-language-related study profiles over the course of about two years are given in Table 1 (lower part). The results supported the (unconstrained) model with varying correlations at T1 and T2 in both groups. Comparing models with and without constrained correlations between listening and reading comprehension to equal parameter values across both studied groups favored the constrained model (AIC = 23,587.90, BIC = 23,703.25, BIC_*adj*_ = 23,617.54; vs. AIC = 23,590.61, BIC = 23,714.52, BIC_*adj*_ = 23,622.47; χ^2^(2) = 1.27, *p* = 0.47). Lower correlations of the PVs were again found at T1 with 0.55 compared to T2 with 0.74, giving evidence that listening and reading comprehension skills converge in upper secondary education in Germany in all studied groups.

## Discussion

In this study, the effects of in- and out-of-school learning opportunities on the associations between both skills in upper secondary education were analyzed from English-language assessment data, which offers a valid and reliable data source to study the interplay of these skills. The results revealed converging receptive skills from the beginning to the end of secondary education in Germany. The finding was robust across different student groups (language-related versus non-language-related study profiles, and students with different levels of self-reported English-language-related extracurricular activities), which were analyzed in order to test the influence of different in- and out-of-school learning activities.

The finding of a growing overlap of listening and reading scores extends results by Tilstra et al. (2009), who examined this effect up to ninth grade (i.e., nearly the end of lower secondary education) as part of a study on the simple view of reading. Substantial differences in the learning gains between both skills were also noticeable in this study. In a contemporary theory of text comprehension Aryadoust (2017) described that listeners are disadvantaged in low-level processes of (word) recognition compared to readers due to the transitory auditory input. Large gains in listening comprehension over two years of upper secondary education, low stability of the proficiency scores (results not presented here; see Leucht et al., 2015) and converging skills illustrate a different level of competence emerging at the end of upper secondary education. The results suggest that modality specificity becomes a less important factor to affect comprehension test scores at the end of secondary education in Germany. In line with the theory of Aryadoust (2017), summarizing these findings may also indicate that difficulties with perceptually earlier, modality-specific processes in ESL learning were simply overcome by a larger group of students over the course of secondary education.

More research is obviously needed to verify this assumption and to trace the effects back to underlying subskills, in particular with experienced ESL learners in higher tracks of education. Recently, Gottardo et al. (2018) “unpacked” listening comprehension by examining the contribution of subcomponents of the skill (vocabulary, morphological awareness, syntax knowledge) to reading comprehension. A growing number of studies also differentiated subcomponents underlying listening and reading comprehension skills (e.g., Song, 2008; Goh and Aryadoust, 2014) by means of psychometric approaches. Research on the development of receptive language skills clearly benefits from a closer look on common subcomponents.

Still, increasing correlations between receptive skills shifts the focus of research more on general text comprehension skills. Previous research demonstrated that different conclusions on the test scores need to be drawn for a general text comprehension dimension compared to modality-specific scores, as text comprehension is, for example, conceptually closer to general cognitive abilities compared to modality-specific processing (Hartig and Höhler, 2008). Even reversed effects of covariates like gender occur when modality-specific processes are partialed out (Hartig and Höhler, 2008). The results presented here revealed a consistent effect across different language-learning groups (with marginally different correlation levels depending on study profiles), which simplifies the interpretation toward a general development in upper secondary education in Germany. Thus, in this age group emphasis may be placed on didactic and educational settings in the future that focus on fostering general comprehension skills instead on modality-specific aspects.

Obviously, the sampling of students from upper secondary education still needs to be considered when interpreting the results. This educated group of students certainly owns specific individual skills such as high (working) memory capacity, which is known to be related to receptive language skills (e.g., Vandergrift and Baker, 2015), but assumedly also differs in terms of individual modality preferences in text comprehension (e.g., Kürschner et al., 2005). Thus, it seems reasonable to additionally study the development of associations between both skills in samples from less institutionalized learning, such as vocational education, adult education, or advanced business English training. It should also be noted that several major transformations have become obvious in terms of media consumption in recent years in Germany. A growing number of online media service providers rely predominantly on younger users and make English-language media with entertainment and academic content more popular in this age group. The effects of these changes in English-language-related activities on the development and the associations of listening and reading comprehension might become fully apparent only over a longer period, studied with multiple cohorts.

## Data Availability Statement

The datasets generated for this study will not be made publicly available. Contact information to request access to the dataset is: IPN – Leibniz Institute for Science and Mathematics Education.

## Ethics Statement

Ethical review and approval was not required for the study on human participants in accordance with the local legislation and institutional requirements. Written informed consent to participate in this study was provided by the participants’ legal guardian/next of kin.

## Author Contributions

CS performed substantial contribution to the conception of the study and the interpretation of the results, conducted the statistical analyses, drafted the manuscript, and approved the submitted version. JF performed substantial contribution to the conception of the study and the interpretation of the results, reviewed the manuscript critically for important intellectual content, and approved the submitted version. ML performed substantial contribution to the conception of the study and the interpretation of the results, reviewed the manuscript critically for important intellectual content, and approved the submitted version.

## Conflict of Interest

The authors declare that the research was conducted in the absence of any commercial or financial relationships that could be construed as a potential conflict of interest.
